# Transactions National Dental Associations

**Published:** 1913-07-15

**Authors:** 


### BIBLIOGRAPHY.

TRANSACTIONS NATIONAL DENTAL ASSOCIATIONS. The proceedings of the Sixteenth Annual Meeting of this society, held at Washington, D. C., September 10 to 13, 1912, are published by the press of the *Dental Cosmos,* in the usual form and with the same care that characterizes the work of this publication. The material has already appeared in the pages of the *Dental Cosmos* and requires no further comment. It is a great convenience to have this material reprinted and bound in book form, so that it may be more readily consulted. We often wonder how many dentists keep files of such publications, or if they do keep them, what use they make of them. Are they ever re-read except for research purposes? They will be of great value as historic material in the future years, perhaps as curiosities.

